# Workplace bullying and violence as risk factors for type 2 diabetes: a multicohort study and meta-analysis

**DOI:** 10.1007/s00125-017-4480-3

**Published:** 2017-11-13

**Authors:** Tianwei Xu, Linda L. Magnusson Hanson, Theis Lange, Liis Starkopf, Hugo Westerlund, Ida E. H. Madsen, Reiner Rugulies, Jaana Pentti, Sari Stenholm, Jussi Vahtera, Åse M. Hansen, Mika Kivimäki, Naja H. Rod

**Affiliations:** 1 Section of Social Medicine, Department of Public Health, University of Copenhagen, Gothersgade 160, 1014 Copenhagen, Denmark; 2 Stress Research Institute, Stockholm University, Frescati hagväg 16, 104 05 Stockholm, Sweden; 3 Section of Biostatistics, Department of Public Health, University of Copenhagen, Copenhagen, Denmark; 4 Center for Statistics Science, Peking University, Beijing, People’s Republic of China; 5 National Research Centre for the Working Environment, Copenhagen, Denmark; 6 Department of Psychology, University of Copenhagen, Copenhagen, Denmark; 7 Department of Public Health, University of Turku, Turku, Finland; 8 Turku University Hospital, Turku, Finland; 9 Finnish Institute of Occupational Health, Helsinki, Tampere and Turku, Finland; 10 Department of Epidemiology and Public Health, University College London, London, UK; 11 Clinicum, Faculty of Medicine, University of Helsinki, Helsinki, Finland

**Keywords:** Bullying, Diabetes, Meta-analysis, Occupational health, Stress, Violence, Workplace

## Abstract

**Aims/hypothesis:**

The aim of this multicohort study was to examine whether employees exposed to social stressors at work, such as workplace bullying and violence, have an increased risk of type 2 diabetes.

**Methods:**

The study included 45,905 men and women (40–65 years of age and free of diabetes at baseline) from four studies in Sweden, Denmark and Finland. Workplace bullying and violence were self-reported at baseline. Incident diabetes was ascertained through national health and medication records and death registers. Marginal structural Cox models adjusted for age, sex, country of birth, marital status and educational level were used for the analyses.

**Results:**

Nine per cent of the population reported being bullied at work and 12% were exposed to workplace violence or threats of violence. Bullied participants had a 1.46 (95% CI 1.23, 1.74) times higher risk of developing diabetes compared with non-bullied participants. Exposure to violence or threats of violence was also associated with a higher risk of diabetes (HR 1.26 [95% CI 1.02, 1.56]). The risk estimates attenuated slightly when taking BMI into account, especially for bullying. The results were similar for men and women, and were consistent across cohorts.

**Conclusions/interpretation:**

We found a higher risk of incident type 2 diabetes among employees exposed to bullying or violence in the workplace. Further research is needed to determine whether policies to reduce bullying and violence at work may reduce the incidence of type 2 diabetes in working populations. Research on the mechanisms is also highly warranted.

**Electronic supplementary material:**

The online version of this article (10.1007/s00125-017-4480-3) contains peer-reviewed but unedited supplementary material, which is available to authorised users.

## Introduction

Recent meta-analyses have suggested that psychosocial work characteristics, such as job insecurity [1] and long working hours [2], are associated with a moderately higher risk of diabetes, while the health effects of highly adverse social work stressors, such as bullying and violence at work, are far less well documented. The prevalence of workplace bullying, defined as persistent, repeated harassing, offending and socially excluding behaviours of psychological nature over a long period [3], ranges from 5% to 24% depending on the country [4]. The prevalence of violence and threats of violence also vary between countries, but are generally more common in occupations with client contact; for example, 19.9% of nurses in the USA have reported exposure to violence and threats of violence at work [5].

Bullying and violence can adversely affect personal resources, such as self-esteem and coping capacity [3]. They have also been linked with an increased risk of chronic conditions, including type 2 diabetes [6], which is characterised by insulin resistance in liver and muscle and progressive beta cell failure [7]. Induced negative emotions, such as depression and anxiety [8, 9], may contribute to diabetes risk [10] through prolonged activation of the hypothalamic–pituitary–adrenal axis and sympathetic nervous system, or indirectly through impaired sleep, for example [11]. Furthermore, stress-related coping strategies, such as comfort eating behaviour with an increased preference for energy and nutrient dense foods [12], may result in weight gain or an increase in waist circumference [13], which are both pivotal risk factors for diabetes [14].

However, it is unknown whether bullying or violence at work affects diabetes risk in the general population. To the best of our knowledge, the only existing study on negative interpersonal relations and diabetes was a cross-sectional study of 8499 male and 9025 female employees in the USA in 2015. It postulated a higher prevalence of type 2 diabetes among employees exposed to workplace bullying, threats or other kinds of harassment compared with those who were not exposed to these social stressors [15]. However, due to the cross-sectional study design, the temporality of the association is unclear. Therefore, the aim of the present study was to assess the prospective relationship between bullying and violence at work and the risk of incident type 2 diabetes using longitudinal individual-level data from four large Nordic cohort studies involving more than 40,000 participants.

## Methods

### Study baseline

The study population was derived from four prospective cohort studies including the Swedish Work Environment Survey (SWES) [16], the Swedish Longitudinal Occupational Survey of Health (SLOSH) [17], the Finnish Public Sector Study (FPS) [18] and the Danish Work Environment Cohort Study (DWECS) [19]. For more details on the individual cohorts, please see the electronic supplementary material (ESM Study populations). Ethical approval was obtained from the Regional Ethical Review Board in Stockholm for SWES and SLOSH, and the Ethics Committee of the Hospital District of Helsinki and Uusimaa for FPS. In Denmark, questionnaire- and register-based studies do not require ethics committee approval. DWECS was approved by and registered with the Danish Data Protection Agency.

**Fig. 1:**
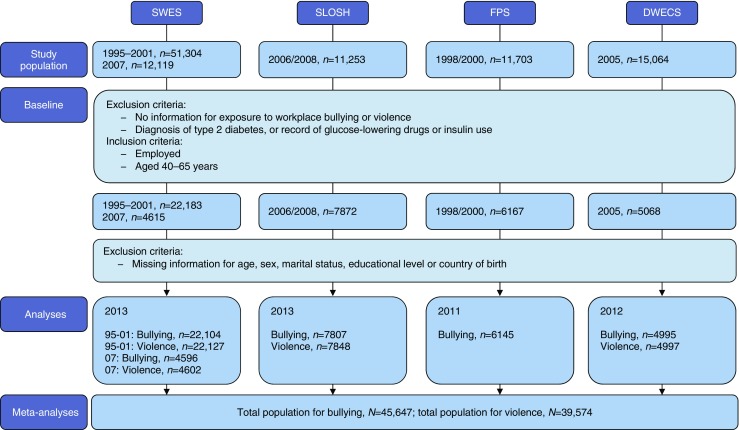
Flow chart of the study population

Our baseline sample was restricted to participants with information on either workplace bullying or violence at baseline (Fig. 1). We only included those who were employed and aged between 40 and 65 years. Participants younger than 40 years of age were excluded in order to minimise outcome misclassification of type 1 diabetes and polycystic ovary syndrome [20]. Those who were previously diagnosed with diabetes or had previously used glucose-lowering medications or insulin were excluded. Our final sample included 19,280 men and 26,625 women.

### Workplace bullying and violence

Being bullied or targeted by violent actions or threats of violence was measured using questionnaires with similar wording in all cohorts. We defined workplace bullying as reporting having been bullied at the workplace at least once during the past 12 months (in SWES, SLOSH and DWECS). In FPS, the time frame was slightly different as participants were asked whether they were currently being bullied. Workplace violence was measured as the experience of having been the target of violent actions or threats of violence in the past 12 months at the workplace (in SWES, SLOSH and DWECS). Violence was not measured in FPS; therefore, this cohort was not included in the analysis of violence. Participants were further identified as ‘frequently exposed’ if the bullying/violence occurred at least once a week. Detailed information on measurements and definitions of workplace bullying and violence can be found in ESM Table 1.

### Type 2 diabetes

Using unique personal identification numbers in Sweden, Finland and Denmark, all participants were linked to nationwide health registers. We used available information for each country and at different historical time points to capture all incidences of diabetes. Type 2 diabetes was identified with codes ICD-8/9 250 and ICD-10 E11 (www.who.int/classifications/icd/en/) from inpatient registers only (in SWES95-01 and FPS) or both inpatient and outpatient registers (in SWES07, SLOSH and DWECS) and combined with information from death registers (in all cohorts). This was supplemented with information on prescription medication using Anatomical Therapeutic Chemical codes A10A, A10B and A10X (in SWES07, SLOSH and FPS). In DWECS, individuals were identified in the Danish Diabetes Register, which combines information from the national patient register on the use of insulin or oral glucose-lowering drugs, registration for chiropody for treatment of diabetes-related complications and individuals with more than five blood glucose measurements within the period of a year [21]. For the individuals receiving insulin treatment, this register includes both type 1 and type 2 diabetes. However, as all participants were free from diabetes at baseline at an age of at least 40 years, type 2 diabetes probably represents the majority of these individuals.

### Other variables

Potential confounders were identified based on prior knowledge and using directed acyclic graphs [22]. In addition to age and sex, potential confounders included country of birth, educational level and marital status. Information on educational level was obtained from the social registers in each country and was categorised as ≤9 years, 10–12 years and ≥13 years. Marital status, as a proxy for social support outside work, was also obtained from the population registers (in SWES, SLOSH and DWECS) or self-reported questionnaires (in FPS). It was categorised as unmarried, married/cohabiting, divorced/separated or widowed. Country of birth was self-reported and classified as ‘Nordic countries’, ‘other European countries’ and ‘other continents’ (in SWES, SLOSH and DWECS). Country of birth was not measured in FPS, but the vast majority of hospital employees in the cohort are from Nordic countries. We assumed that mental illness, excessive alcohol consumption and obesity would be on the causal pathway from bullying or violence to type 2 diabetes (and thus should not be controlled for). However, mental illness, excessive alcohol consumption and obesity may also be causes of workplace bullying and violence, and thus confounders. Therefore, we chose to include adjustment for BMI, alcohol consumption and mental illness in sensitivity analyses. BMI (in SLOSH, DWECS and FPS) was calculated using self-reported height and weight, grouping according to the WHO categories: underweight (<18.5 kg/m^2^), normal (18.5–24.9 kg/m^2^), overweight (25–29.9 kg/m^2^) and obese (≥30 kg/m^2^). Information for alcohol consumption was available in FPS, DWECS and SLOSH. Alcohol consumption was divided into ‘risky’ or ‘non-risky’ based on exceeding or not exceeding 16/24 (in FPS) or 14/21 (in DWECS and SLOSH) alcohol units (12 g of alcohol per unit) per week for women/men, or weekly consumption of six or more units per occasion (in SLOSH). Mental illness was identified using only inpatient registers (in SWES95-01 and FPS) or both inpatient and outpatient registers (in SWES07, SLOSH and DWECS), and dichotomised into having at least one mental illness problem and having no mental illness. Furthermore, workplace violence is very likely to be clustered in occupations with frequent client contact. Thus, we separated personal and protective service workers, healthcare professionals, social work professionals and teaching professionals from other occupations using the current Swedish and Danish adapted version of the International Standard Classification of Occupations (ISCO-88) following suggestions from Madsen et al [23].

### Statistical analysis

Data from the individual cohorts were analysed separately. The cohort-specific results were later combined using meta-analyses. Two datasets with slightly different numbers of participants were created for each cohort for the analyses of bullying and violence, excluding participants with information missing for any of the covariates (Fig. 1). For the sensitivity analyses including SLOSH, FPS and DWECS, participants with missing information for BMI (in the BMI-adjusted analyses) or both BMI and alcohol consumption (in the BMI- and alcohol-adjusted analyses) were excluded.

For the main analyses we applied a marginal structural Cox model estimated by using inverse probability (IP) weights [24]. This approach is based on a counterfactual framework. Given the properly identified confounders, the method provides an estimate of the marginal HR. This corresponds to comparing the risk of diabetes in a pseudo population where everyone is bullied with the same population where everyone is not bullied. Our main analyses were conducted in three steps. In step one, the stabilised IP weight was obtained for each individual included by fitting a logistic model for the conditional probability of being exposed based on relevant confounders in each analysis [24]. In this step, the positivity assumption was verified in all of the analyses. Step two was to fit weighted Cox proportional hazard models using age as the underlying timescale. The IP weights varied depending on whether the Cox proportional hazard models were age- and sex-adjusted or fully adjusted. The proportional hazards assumption was tested using log (-log(survival)) curves. If a result was doubtable, a stratified Cox model was performed to confirm the violation. In our study, none of the violations were of major concern. In step three, the robust confidence interval was calculated using standard errors generated from bootstrapping steps one and two a total of 500 times.

Sensitivity analyses based on specific IP weights suitable for each analysis were conducted on: (1) sex-stratified analyses; (2) analyses adjusting for BMI; (3) analyses adjusting for both BMI and alcohol consumption; (4) analyses adjusting for mental illness, in addition to the main adjustments; (5) analyses only including the first 4 years of follow-up in order to test whether the effect was dependent on length of follow-up; (6) analyses considering a one year washout period to address the possibility of reversed causality; (7) analyses based on different definitions of diabetes (inpatient plus death vs inpatient plus medication plus death vs inpatient plus outpatient plus death); (8) analyses to test a potential dose–response relationship between frequency of exposure to bullying/violence and risk of type 2 diabetes in cohorts with available information on frequency of exposure (in SLOSH and SWES); and (9) further stratified analysis for violence towards individuals in occupations with frequent client contact (in SLOSH, SWES and DWECS).

To adjust for the small number of studies included, the risk estimates from each cohort were combined in the fixed-effect meta-analyses [25]. The *I* ^2^ statistic was used to test for heterogeneity between the study-specific estimates. All tests of statistical significance were two-sided and the significance level was set at 0.05 using SAS version 9.4 (SAS Institute, Cary, NC, USA) and R package ‘meta’ version 4.8-2.

## Results

### Baseline characteristics

Nine per cent of the participants reported exposure to bullying at work (Table 1). The prevalence of bullying varied between studies, with the highest prevalence observed in SLOSH (13%). The combined analysis on workplace bullying included 45,647 participants (26,396 women and 19,251 men).

**Table 1:** Summary of the studies that provided individual participant data used in the analyses of the associations of type 2 diabetes with workplace bullying and violence

Study	Country	Baseline	Women (%)	Mean age (SD)	Mean follow-up length (years)	Bullying (%)	Violence (%)	Type 2 diabetes incidence^a^
SWES95-01	Sweden	1995–2001	53	50 (6.3)	15.1	8	11	8.5
SWES07	Sweden	2007	53	51 (7.0)	6.1	9	14	46.5
SLOSH	Sweden	2006/2008	55	52 (7.0)	7.0	13	17	52.4
FPS	Finland	1998/2000	88	49 (5.7)	12.8	8	–	37.8
DWECS	Denmark	2005	51	50 (6.3)	6.9	9	7	60.3
Total		1995–2008	58	50	11.7	9	12	23.0

^a^Per 10,000 person-years

All numbers were calculated based on the largest available sample

Twelve per cent of the participants had experienced violence or threats of violence at work within the past 12 months (Table 1). This proportion varied between the cohorts, with the highest prevalence reported in SLOSH (17%) and the lowest in DWECS (7%). There was no information on workplace violence reported for FPS. Therefore, the analyses on workplace violence were based on the 39,574 participants from the remaining three cohorts, including 21,023 women and 18,551 men. The prevalence of violence varied greatly between different occupations. The highest prevalence of violence or threats of violence were found among occupations with frequent client contact, including social work professionals (>46%), personal and protective service workers (>29%), healthcare professionals (>25%) and teaching professionals (>16%).

Only 2–4% of participants reported exposure to both workplace bullying and violence, and there was very little statistical agreement between the two measures across all the included cohorts (Cohen’s κ < 0.20).

### Workplace bullying and type 2 diabetes

**Fig. 2:**
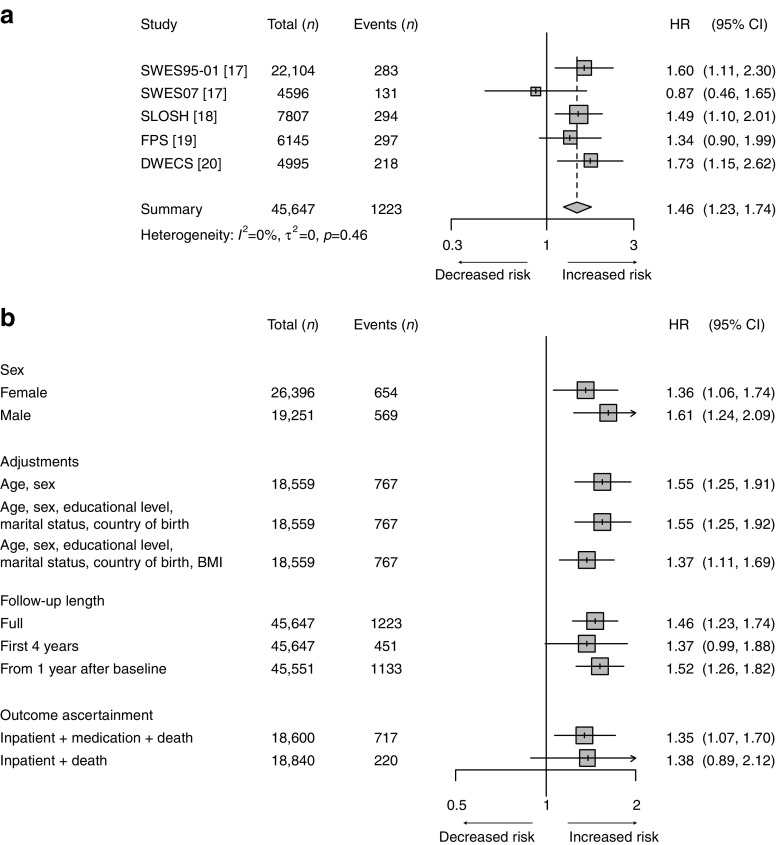
(**a**) Main analysis using a fixed-effect model on the association of workplace bullying with type 2 diabetes, after adjustment for age, sex, educational level, marital status and country of birth. (**b**) Sensitivity analysis using a fixed-effect model by sex, adjustments (based on SLOSH, FPS and DWECS), follow-up time (based on SLOSH, FPS and SWES07) and case ascertainment

During a mean follow-up of 11.7 years, we identified 1223 incident cases of type 2 diabetes. After adjustment, being bullied at work was associated with a higher risk of type 2 diabetes (HR 1.46 [95% CI 1.23, 1.74]; Fig. 2). The risk estimates varied across the studies but, apart from a very unstable estimate from SWES07 (based on only 131 incident type 2 diabetes events), they were all in the same direction and there was no indication of heterogeneity (*I* ^2^ < 0.1%). The observed associations were similar for men (HR 1.61 [95% CI 1.24, 2.09]) and women (HR 1.36 [95% CI 1.06, 1.74]). Information on BMI was only available in SLOSH, FPS and DWECS. After additional adjustment for BMI, the risk estimate for workplace bullying on type 2 diabetes attenuated from 1.55 (95% CI 1.25, 1.92) to 1.37 (95% CI 1.11, 1.69). Additional adjustments for alcohol consumption did not change the risk estimate (ESM Fig. 1). Adjustment for mental illness also did not change the risk estimate (ESM Fig. 2). Further, when limiting our analysis to the first 4 years of follow-up the risk estimate remained similar to that of the main analysis (HR 1.37 [95% CI 0.99, 1.88]). In addition, a one year washout period or differences in case ascertainment across the studies did not change the risk estimate (Fig. 2).

The two Swedish cohorts (SWES and SLOSH) provided information on the frequency of bullying. Of those who were bullied, 10% reported being bullied frequently (i.e. at least once a week) and 90% reported being bullied occasionally. Compared with those who had not experienced bullying in the past 12 months, those who had experienced bullying occasionally had a higher risk of type 2 diabetes (HR 1.57 [95% CI 1.23, 1.99]), whereas those who experienced bullying frequently did not have a clear excess risk of type 2 diabetes (HR 1.24 [95% CI 0.58, 2.64]) (based on only seven incident cases of type 2 diabetes in the frequently bullied group).

### Workplace violence and type 2 diabetes

**Fig. 3:**
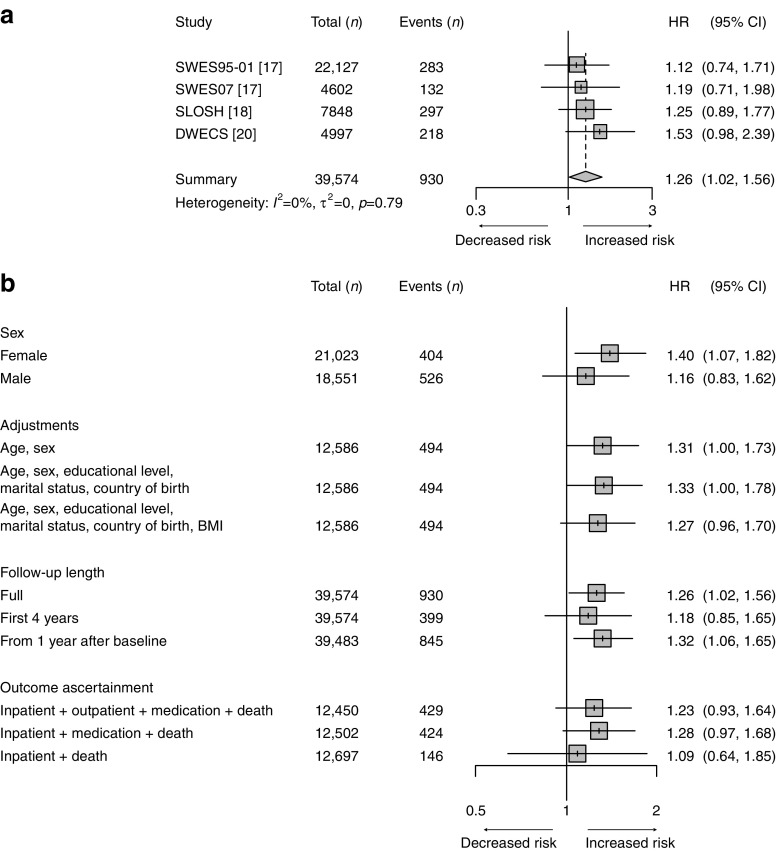
(**a**) Main analysis using a fixed-effect model on the association of workplace violence with type 2 diabetes, after adjustment for age, sex, educational level, marital status and country of birth. (**b**) Sensitivity analysis using a fixed-effect model by sex, adjustments (based on SLOSH and DWECS), follow-up time (based on SLOSH and SWES07) and case ascertainment

During a mean follow-up of 11.4 years, we identified 930 incident cases of type 2 diabetes in the three cohorts used for the analyses on violence. After adjusting for age, sex, educational level, marital status and country of birth, workplace violence or threats of violence were associated with a higher risk of type 2 diabetes (HR 1.26 [95% CI 1.02, 1.56]) (Fig. 3). The risk estimates varied slightly between the different studies but there was no indication of heterogeneity across the studies (*I* ^2^ < 0.1%) (Fig. 3). In addition, no obvious differences between men and women were revealed. Additional adjustment for BMI slightly changed the risk estimate from 1.33 (95% CI 1.00, 1.78) to 1.27 (95% CI 0.96, 1.70). Further adjustments for alcohol consumption and mental illness did not affect the risk estimate (ESM Figs 1, 2). Moreover, the risk estimate was not influenced by the follow-up length (Fig. 3). The test for different outcome ascertainment showed no difference using various case ascertainment methods (Fig. 3).

In the analysis of the dose–response relationship (based on SLOSH and SWES), among those experiencing violence at work, 17% were targeted by workplace violence frequently (at least once a week; 12 incident cases of type 2 diabetes in this group) and 83% were occasionally exposed (68 incident cases of type 2 diabetes in this group). Neither occasional (HR 1.18 [95% CI 0.94, 1.50]) nor frequent exposure to violence (HR 0.95 [95% CI 0.52, 1.76]) was clearly associated with an increased risk of type 2 diabetes.

In addition, we observed a similar excess risk for occupations with (HR 1.20 [95% CI 0.90, 1.60]) and without frequent client contact (HR 1.36 [95% CI 1.03, 1.81]).

## Discussion

In this large multinational, multicohort study, approximately one in ten employees reported being exposed to bullying or violence/threats of violence at work. Both men and women who were exposed to these severe social stressors were at a higher risk of developing type 2 diabetes. The higher risk was consistent across cohorts and independent of follow-up length or the method of case ascertainment. When we adjusted for BMI, the associations were attenuated but remained statistically significant in the case of bullying and suggestive in the case of violence. These findings add to the previous cross-sectional study by Khubchandani et al that reported a risk estimate (OR 1.48 [95% CI 1.03, 2.15]) that is similar to our findings but is based on a less specific definition of bullying that included being harassed, threatened or bullied any time in the past 12 months [15].

We did not find a dose–response relationship trend for workplace bullying. The point estimate for those being frequently bullied was lower than those being occasionally bullied. This may be due to a very limited number of incident diabetes events in the frequently bullied group generating a very wide confidence interval. However, we did not find a dose–response relationship for workplace violence either. Occupations with frequent client contact can be a proxy of frequent workplace violence [23]; however, the point estimate was similar between occupations with frequent client contact and those without. A possible explanation for the lack of a dose–response relationship here could be a protective effect from general expectations and/or training prior to workplace violence among employees in occupations with frequent client contact [26]. It may also be attributable to cognitive adaption including finding meaning, enhancing self and gaining mastery [27] following frequent exposure.

### Bullying vs violence

Our results suggest that while both bullying and violence represent negative interpersonal relationships they most probably constitute different concepts, with only 2–4% of participants reporting being exposed to both, and very poor statistical agreement indicating that bullying and violence are two distinct social stressors. Harassment and bullying refer to psychological aggression, including behaviours such as unfair criticisms, humiliating work tasks, isolation, ignorance and spreading rumours [3]. Violence or threats of violence on the other hand are more likely to be understood as physical violence or verbal threats relating to physical violence [28], and the actions included can be exemplified as pushing, kicking and screaming [29]. There can be situations where behaviours displaying bullying and violent characteristics overlap, especially when the negative behaviour of concern has been observed to persist and be repeated over a long period [3]. However, in most situations, bullying at work is often characterised by negative behaviours from colleagues and supervisors, sometimes also from clients [30], whereas the overwhelming proportion of violence at work is derived from clients, students, customers, patients, etc [29]. Hence, workplace bullying and workplace violence seem to be distinct behaviours and, consequently, their induced emotions can be different. However, the associated behavioural appraisals and physiological reactions may be similar and may explain the comparable associations with risk of diabetes observed in the present study.

### Plausible pathways

Being bullied is regarded as a severe social stressor that may activate the stress response system and lead to a range of downstream biological processes that may contribute towards the risk of diabetes [31]. In agreement with this, bullying at work has, for example, been found to be related to a higher level of saliva dehydroepiandrosterone sulphate [32], although no increase in saliva cortisol has been documented [33] and there is no clear finding from longitudinal studies on stress biomarkers [34]. Nevertheless, these hormones may work together in affecting cellular activities and metabolic, cardiovascular and immune variables [15, 35]. Metabolic changes and obesity are also possible mechanisms underlying the observed higher risk of type 2 diabetes associated with both bullying and violence, as stress responses may be related to the endocrine regulation of appetite [36]. In the present study, the relationships between bullying and violence and type 2 diabetes attenuated after adjustment for baseline BMI, which can either be due to the fact that obese employees are more likely to be targets for workplace bullying or violence or that exposed employees are more likely to gain weight and become obese. The first explanation (i.e. that obesity leads to bullying) was, however, not supported in a sensitivity analysis presented in a previous paper on bullying and cardiovascular disease based on data from the FPS study, where the authors did not find an association between baseline BMI and incident workplace bullying [37]. On the other hand, it is likely that both workplace bullying and violence can induce comfort eating behaviour [12] or increase the risk of experiencing negative emotions [8, 26, 38], and further contribute to weight gain and subsequent development of type 2 diabetes, making the causal pathway very plausible.

### Methodological considerations

The exposure measurements differed slightly between the studies, especially the measurements from FPS, as in this study bullying is defined as ‘currently being bullied’ rather than bullying experienced in the past 12 months. Furthermore, workplace bullying was self-reported without providing a definition, which did not reflect the formal definition of persistent or repeated events [3]. According to a meta-analysis on measurement of workplace bullying in Nordic countries [4], the provision of a definition did not impact prevalence, presumably because the concept of bullying at schools and workplaces is well established. However, in this study, given the subjectivity of solely using a self-reporting method, it is possible that our results are, to some extent, affected by exposure misclassification. Furthermore, workplace bullying and violence were only measured at baseline, ignoring the possibility of changes in exposure status over time [39], which may have diluted our results.

The incidence of type 2 diabetes was smaller in SWES95-01 compared with the other cohorts. This difference may be mainly ascribed to the fact that SWES95-01 could not be linked to the medication register. However, a meta-analysis is a robust tool with which to incorporate such variations. It is reassuring that although the association in FPS is possibly diluted by using a narrower exposure window it showed a similar risk estimate and direction as the other cohorts.

Type 2 diabetes was ascertained differently across countries and at different historical time points, leading to some degree of misclassification. We chose to use the most comprehensive definition in each study to reduce outcome misclassification, which came at the cost of direct comparability in incidence rates across studies. However, when standardising case ascertainment across the studies in the sensitivity analyses, we found no obvious heterogeneity for the risk estimates dependent on case ascertainment method, suggesting that such misclassification is not a major source of bias. In addition, we have considered several important confounders. However, in observational settings, unmeasured confounders are unavoidable, e.g. personality and genetic factors. Thus, our results should be interpreted with caution.

To the best of our knowledge, no previous longitudinal studies have addressed the relationships between workplace bullying and workplace violence and type 2 diabetes. All the analyses were done under the counterfactual framework, ensuring a more straightforward interpretation of the results. Further, we applied the best available outcome measurement, linking to nationwide registries to allow for a nearly complete follow-up and to minimise misclassification of other diseases. Our large sample size and long follow-up period provided sufficient statistical power to assess total and sex-specific effects, and to conduct relevant sensitivity analyses adjusted for different variables and case ascertainment.

In conclusion, we have shown a moderate and robust association between workplace bullying and violence and the development of type 2 diabetes. Both bullying and violence or threats of violence are common in the workplace. Research on bullying and violence prevention policies with workplaces as the target are warranted to determine whether these policies could be effective means of reducing the incidence of type 2 diabetes. Further study of possible pathways, for example through weight gain, negative emotions and the physiological stress response, will be crucial in providing an understanding of the causal mechanisms, as well as developing more cost-effective interventions with surrogate outcomes.

## Electronic supplementary material

ESM (PDF 145 kb)

## Abbreviations

DWECS: Danish Work Environment Cohort Study
FPS: Finnish Public Sector Study
IP: Inverse probability
SLOSH: Swedish Longitudinal Occupational Survey of Health
SWES: Swedish Work Environment Survey
