# Supplementary material for: Workplace bullying and violence as risk factors for type 2 diabetes: a multicohort study and meta-analysis
Source: Diabetologia. 2017 Nov 13;61(1):75–83. doi: 10.1007/s00125-017-4480-3 (PMC6005336; doi:10.1007/s00125-017-4480-3)
Supplement: Supplementary file 1 — (PDF 145 kb) [file 125_2017_4480_MOESM1_ESM.pdf]

Electronic supplementary material (ESM)

ESM Study populations

Swedish Work Environment Study (SWES) is a nationally representative survey commissioned by the Swedish Work Environment Authority every second year since 1989, covering a sub-population from the Labor Force Survey [1]. Participants were interviewed by telephone, supplemented with a mailed questionnaire. We used five survey waves from the years 1995, 1997, 1999, 2001 and 2007. These waves were chosen because of the availability of health data and exposure information on workplace bullying and violence. Wave 2003 and wave 2005 had the same participants as SLOSH 2006 and 2008. Therefore, they were not included. To make full use of the register-based information, SWES was divided into two sub-cohorts for the analyses, SWES95-01 representing the surveys from 1995 to 2001 and SWES07 representing the survey from 2007.

Swedish Longitudinal Occupational Survey of Health (SLOSH) was established in March 2006 based on the respondents from SWES of 2003 [2]. The questionnaire had two versions, one for those gainfully employed for more than 30% at the time of response and one for all others. In 2008, additional individuals from SWES 2005 were added to the cohort. We used the baseline for those participating in SLOSH in 2006 or newly recruited in 2008, who were gainfully employed. The response proportions for SLOSH were 65% in 2006 and 61% in 2008.

Finnish Public Sector Study (FPS) consisted of employees in the municipal services of 10 Finnish towns and 21 public hospitals, aged between 18 and 65 years. Our baseline population included hospital employees who responded to the survey in 1998 or 2000 and based on the employers' records had had a job contract for a minimum of 6 months (response proportion: 73% in 1998 and 67% in 2000) [3]. For participants who responded to both surveys, we used data from the first survey only.

Danish Work Environment Cohort Study (DWECS) is a cohort study based on a random sample from the Central Population Register of Denmark in 1990 for people aged between 18 and 69 [4]. The study was initiated in 1990, with follow-ups conducted every 5 years with some extensions over the years. The 4th wave of the study, in 2005, included detailed information on negative interpersonal relationships at work. The overall response proportion was 63%.

ESM Table 1. Measurements for workplace bullying and violence in the three cohorts at the baseline.

	SLOSH (2006/2008)<br>SWES (1995-2001/2007)	FPS (1998/2000)	DWECS (2005)
**Bullying**			
Measurement	Are you subjected to personal persecution in the form of unkind words or behavior from your superiors or fellow workers?	Workplace bullying refers to a situation where someone is subjected to social isolation or exclusion, his or her work and efforts are devalued, he or she is threatened, derogatory comments are made about him or her in his or her absence, or other negative behavior that aims to torment, wear down, or frustrate the victim occurs. Have you been subjected to such bullying?	In the last 12 months, have you been subjected to uncomfortable bullying in your work place?
Definition	Bullying has occurred at some point in the past 12 months.	Answering ’yes, currently’ to the question.	Answering ’yes’ to at least one question.
**Violence**			
Measurement	Are you exposed to violence or the threats of violence in your work?	-	In the last 12 months, have you been subjected to violent threats in your work place?<br><br>In the last 12 months, have you been subjected to physical violence in your work place?
Definition	Exposure to violence or the threats of violence has occurred at some point in the past 12 months.	-	Answering ’yes’ to at least one question.

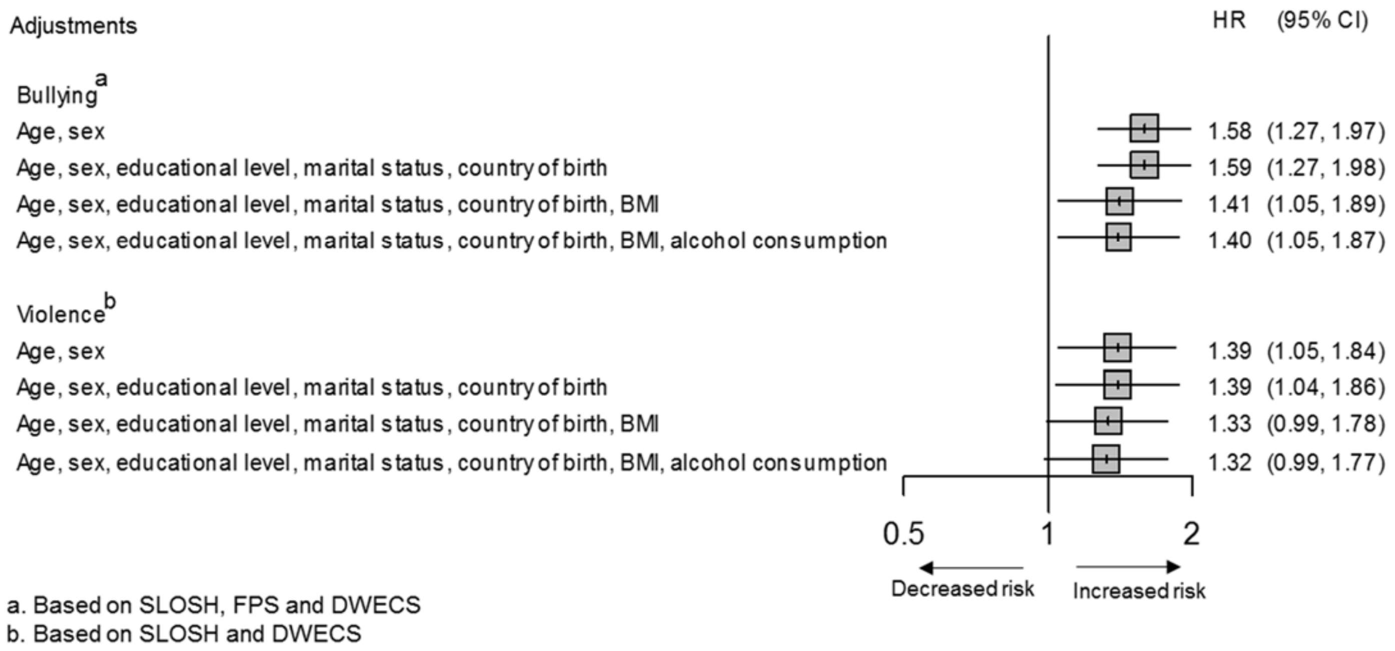

ESM Fig. 1. Adjustments of body mass index (BMI) and alcohol consumption for the association between workplace bullying, violence and type 2 diabetes.

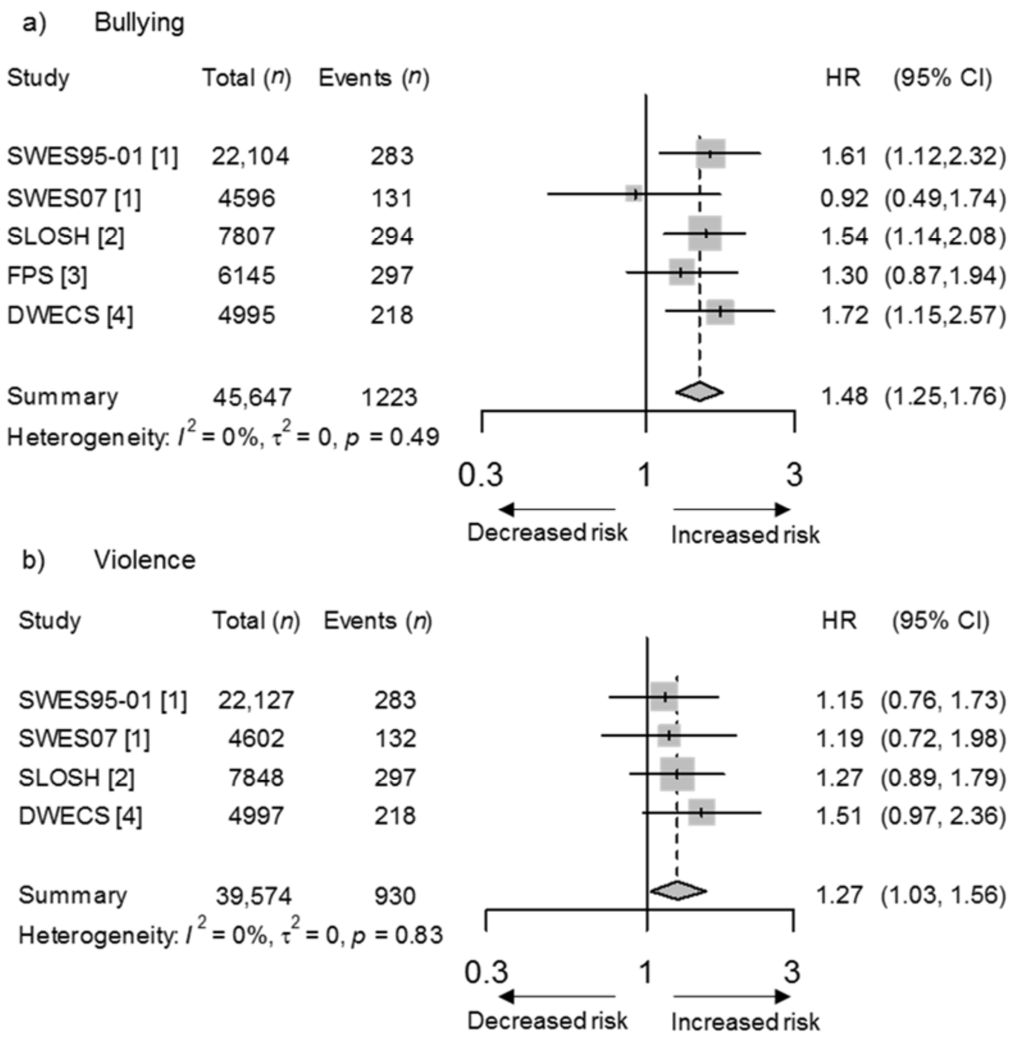

ESM Fig. 2. Further adjustment of mental illness for the association between workplace bullying, violence and type 2 diabetes.

References

[1] Statistic Sweden (2016) Arbetsmiljön 2015 [The working environment 2015]. Available from https://www.av.se/globalassets/filer/statistik/arbetsmiljon-2015/arbetsmiljostatistik-arbetsmiljon-2015-rapport-2016-2.pdf

[2] SLOSH (2017) The Swedish Longitudinal Occupational Survey of Health. Available from http://www.idear-net.net/slosh/in-english/about-the-study

[3] Joensuu M, Kivimäki M, Pentti J, Virtanen M, Väänänen A, Vahtera J (2014) Components of job control and mortality: the Finnish Public Sector Study. Occupational and environmental medicine: 536-542

[4] Burr H, Bach E, Gram H, Villadsen E (2006) Arbejdsmiljø i Danmark 2005: Et overblik fra den NAtionale Arbejdsmiljøkohorte. Available from http://www.arbejdsmiljoforskning.dk/upload/nak2005_arbejdsmiljoe.pdf
